# Field Screening of Rice Germplasm (*Oryza sativa* L. ssp. *japonica*) Based on Days to Flowering for Drought Escape

**DOI:** 10.3390/plants9050609

**Published:** 2020-05-11

**Authors:** Muhammad Shafiq Ahmad, Bingrui Wu, Huaqi Wang, Dingming Kang

**Affiliations:** Department of Plant Genetics and Breeding, College of Agronomy and Biotechnology, China Agricultural University, Beijing 100193, China; ahmad@cau.edu.cn (M.S.A.); wubingrui@mengniu.cn (B.W.)

**Keywords:** trait, terminal drought stress, rainout shelter, rice genotype, plant height, cluster

## Abstract

Terminal drought stress is one of the restrictive factors in rice production and is expected to upsurge under the current situation of climate change. The study evaluated the performance of 2030 rice genotypes under continuous drought stress conditions based on days to flowering (DF). The genotypes under augmented randomized complete block design were sown in May/June of 2017 and 2018 in the field with movable rainout that resulted in huge genetic diversity among the accessions. Descriptive statistics confirmed clear variation among accessions on growth duration, plant height to leaf, plant height to panicle, and germination percentage. Correlation, chemometric, and agglomerative hierarchical cluster analyses were performed that categorized the germplasm into 10 groups. Genotypes in clusters VIII and IX (drought-resistant) revealed better agronomic performance in terms of reduced days to flowering, but conversely taller plant height and higher maturity (%) under severe stress. Genotypes in clusters IV, V, and X were discovered to be drought-susceptible. The screened genotypes like Longjing 12, Longdun 102, Yanjing 22, Liaojing 27, Xiaohongbandao, Songjing 17, and Zaoshuqingsen can be utilized in rice breeding improvement programs for drought tolerance in terms of severe continuous drought, as well as terminal drought stress.

## 1. Introduction

Genebanks are repositories of the variety of crops around the world, and their collections serve as great prospective sources of stress tolerance. The rice genebank at the Institute of Crop Sciences, Chinese Academy of Agricultural Sciences (CAAS), maintains several rice genetic resources, primarily breeding materials or landraces from *Oryza sativa*, *Oryza glaberrima*, and wild and representative species of the *Oryza* genus. CAAS genebank ensures the survival and continuous supply of rice-enhancing genetic resources. Future plant enhancement is based on the genetic variability from conventional varieties and associated wild species to deal with the many abiotic and biotic stresses that jeopardized rice production around the globe [1]. To research the genetic traits that have the potential to withstand various stresses’ effects on rice growth and production, it is, therefore, advisable to explore the extensive array of genetic diversity of rice at the CAAS genebank that provides an admirable chance to identify genotypes for stress acclimatization.

At present, rice (*Oryza sativa* L.) is one of the prominent cereal crops serving more than three billion people. Modeling simulations estimate that agricultural production will need to double by 2050 to sustain the growing population [2]. Rice is cultivated mainly under well-watered ecotypes, consuming 24% to 30% of the total freshwater available for agriculture [3]. Increasing demand for freshwater to meet increasing urban needs would restrict freshwater supplies, threatening global rice production due to limited water tolerance [4]. On the other hand, drought stress is currently a major constraint limiting the production of rice in approximately 23 million ha of rainfed rice grown worldwide [5]. Furthermore, the impact of climate change is already being observed as increased unusual weather patterns are resulting in more regular and severe drought stress events [6]. As projected by global climate models, recurrent drought events under future climate change may result in increased losses when droughts overlap with sensitive stages of crop growth [7]. Rice’s vulnerability to stress from drought is well known during various stages of developmental growth. The major impact of drought stress, however, occurs at flowering and grain filling phases, leading to a significant yield penalty [8].

The effectiveness of a specific method of screening for drought depends on the refinement of genotypes and traits which reflects that of the objective conditions. Lafitte et al. [9] promoted the use of controlled habitats at research stations to make huge developments in choosing drought-tolerant rice varieties. The application of a uniform, repeatable, and controlled stress environment can be achieved more readily in the dry season, and this may maximize the genetic component of the observed variation that assists increasingly available phenotyping skills for identifying and screening rice genotypes against stress.

Sustained breeding efforts achieved significant improvements in developing drought-tolerant rice cultivars. However, progress is slow because of the complexity of the traits involved, the unpredictable occurrence of drought, and limited information on effective screening techniques [5]. Despite the progress made, crop improvement programs cannot be used as immediate measures to reduce the rice yield losses resulting from drought stress. Hence, there is a need for more sources of drought tolerance to cope with a reduction in yield in a variety of various water-deficient conditions. While grain yield is widely used as a selection criterion, several input capitals may be required to accurately determine grain yield for a large-scale germplasm and, thus, for a breeding program, secondary selection may be more effective. This will be possible if we can define a trait that is vastly inherent and linked to the genetic variation in grain yield which can be calculated with scarce resources. One of the well-heritable attributes related to drought tolerance in rice is the postponement of heading under drought stress [10] and can be used to predict genotypic success in drought-prone lowland rainfed areas [11].

Even though selection seems advantageous for multi-stage and intermittent drought [12,13,14], the fate of the chosen germplasm under continuous drought still needs to be explored. Moreover, the assessment of days to flowering (DF) under continuous drought conditions is crucial to the identification of genotypes that would be beneficial for rice-growers facing intermittent stress of water deficiency in certain years and continuous stress in others. The current study aimed to identify and screen 2030 rice genotypes for drought tolerance, particularly delay in flowering under continuous drought stress and its association to flowering duration under terminal drought environments. Finally, we selected 235 genotypes by implementing cluster analysis, which were subsequently screened further to get seven elite genotypes with reduced DF.

## 2. Results

### 2.1. Meteorological Conditions and Drought Stress

**Figure 1:**
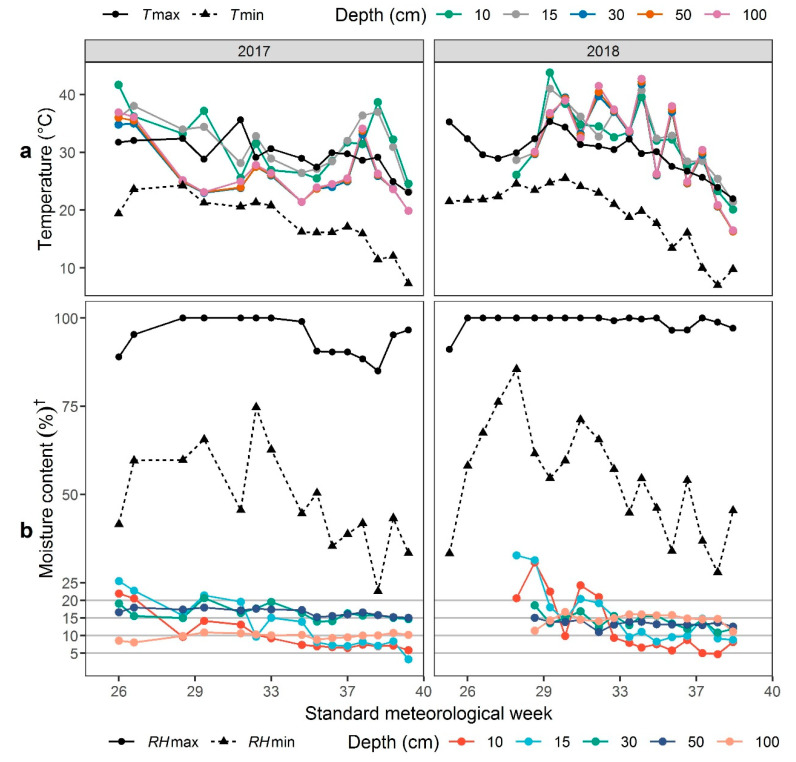
Meteorological and soil conditions at Shangzhuang Agricultural Research Station, Beijing, China from May to October for two consecutive trials in 2017 and 2018: (**a**) air minimum (*T*min) and maximum temperatures (*T*max; °C) at 2 m height from ground level together with soil temperatures (°C) at 10 cm, 15 cm, 30 cm, 50 cm, and 100 cm depths; (**b**) volumetric soil moisture content (%) at soil depths of 10 cm, 15 cm, 30 cm, 50 cm, and 100 cm accompanied by maximum and minimum relative humidity (%) of 2 m height from the ground. *RH*max = maximum relative humidity, *RH*min = minimum relative humidity. ^†^ While reading the *y*-axis of Figure 1b, the moisture content for *RH* is simply moisture content, whereas, for that of various soil depths, it is volumetric soil moisture content.

The selected weather and soil conditions during the trial seasons of three consecutive years (2017–2018) are represented in Figure 1, where time is expressed as standard meteorological weeks (SMW). In this study, total precipitation of upland experimental area during rice-growing season was 483.1 mm in 2017, but we did not move to a rainout shelter until leaf stage three and the field got rainfall of 6.1 mm. Therefore, the total estimated water supply including irrigation and rainfall provided to the crop was 81 mm to 111 mm, which is much lower than the total water use of aerobic rice including irrigation and precipitation (750 mm to 1400 mm) [15]. In 2018, the water supply was more or less same as the first year. Readings recorded at 10 cm, 15 cm, and 30 cm of soil depth were more important concerning the root zone of rice. Recordings at 50 cm and 100 cm were for reference and expressed a comparatively stable trend. The mean minimum to maximum temperatures (*T*min and *T*max) in 2017 and 2018 of crop seasons ranged from 15.01 °C to 27.76 °C and from 15.24 °C to 27.77 °C, respectively (Figure 1a). Soil temperature was generally a little lower than the maximum air temperature in 2017 while a bit higher than it in 2018. Focusing on the root zone of rice at 15 cm depth, the average soil temperature recorded was 31.58 °C and 32.50 °C in 2017 and 2018, respectively, while minimum and maximum soil temperatures for the same depth were 23.2 °C and 38.03 °C in 2017 and 21.43 °C and 41.03 °C in 2018. The average minimum and maximum relative humidity (*RH*min and *RH*max) ranged from 48% to 94.66% and from 54% to 98.91% one-to-one (Figure 1b).

According to our observations over many years, when the topsoil (depth 15 cm) is saturated, the volumetric soil moisture content is generally above 35% to 40%. When it is lower than 20%, the ground begins to crack, and some of the top rice leaves begin to die. When the soil volumetric moisture content is lower than 15%, the ground cracks become obvious, and most leaves show complete or partial death. Finally, when this content is less than 10%, the whole plant suffers severe loss in growth. Therefore, in general, volumetric soil moisture content ranges of 25% to 20%, 20% to 15%, 15% to 10%, and less than 10% in the topsoil indicate “mild”, “moderate”, “severe”, and “extremely severe” soil drought stress, respectively. At our study site, data showed that the incidence of drought initiated (10 cm and 15 cm depth) during the 27th and 30th SMW at the vegetative phase in 2017 and 2018, respectively (Figure 1b). Although minor ups and downs were marked, an overall decreasing trend of volumetric soil moisture content was recorded during both consecutive years. In 2017, at 29th SMW and from 32nd SMW until harvesting, volumetric soil moisture content went down below 10% at 10 cm of soil depth with severe drought occurrence, while the same was observed at 32nd SMW and from 35th SMW to harvesting at a soil depth of 15 cm. Even at harvesting, extremely severe drought was evident. During the second year of study, obvious drought was observed in the field at 31st SMW with soil moisture less than 10% and 15% for 10 cm and 15 cm of soil depths. On the other hand, at 34th SMW, severe drought stress affected the crop that prevailed to end of the growing season. The trend of recordings was consistent with those observed under drought experiments at Shangzhuang Research Station in the summer of 2015 and 2016 by Zu et al. [16].

### 2.2. Variation of Days to Flowering among Treatments, Genotypes, and Controls

The results of the statistical analysis of the effects of treatments, genotypes, and controls on the time to flowering in 2017 and 2018, as well as the pooled data, are summarized in Table 1. Significant differences in all effects were observed for all traits except for block (eliminating treatments) and treatment (test vs. control) in 2017. Table 2 showed descriptive statistics and post hoc mean comparison analysis (HSD) at a 5% level of significance that was performed after augmented randomized complete block design (ARCBD) analysis for days to flowering of control and test genotypes. Test and control entries denoted significant differences in time to flowering. Genotypes revealed high heritability of 94.61% and 90.42% in 2017 and 2018, respectively, which was markedly higher than controls (67.45% in 2017 and 21.35% in 2018). On average, genotypes had a higher number of days to flowering than controls during both growing seasons. Similarly, more comparative pooled variation (standard deviation (*SD*) = 18.8, coefficient of variation (*CV*) = 21.83%) for the same trait was seen among genotypes in comparison with the control group (*SD* = 6.98, *CV* = 8.54%). Control Handao 277 was top-ranked in the control group with the shortest mean time to flowering (75.4 and 77.88 days) in two repeated years of study. B₁ was accredited with average performance in 2017 with 84 mean days to flowering. However, it showed the longest flowering time of 81.92 days to flowering in 2018. As expected, the susceptible control was recognized as having the longest average flowering duration among its counterparts (91.1 days) in 2017 but showed medium performance with mean days to flowering of 81.32 in 2018. Yet, this duration was a bit smaller than that of B₁ in 2018. In 2017, the least variation was expressed by 297-28 (*SD* = 3.66) followed by Handao 277 (*SD* = 5.68) and B₁ (*SD* = 8.01). Contrary to this, 297-28 was attributed with the highest variation (*SD* = 6.4), followed by B₁ (*SD* = 3.33) and Handao 277 (*SD* = 2.56). After summing up all analyses, it was concluded that Handao 277 was a top-ranked control, B₁ was in second position, and 297-28 finished last concerning that flowering period. 

### 2.3. Population Distribution and Genetic Variation among Measured Traits

**Figure 2:**
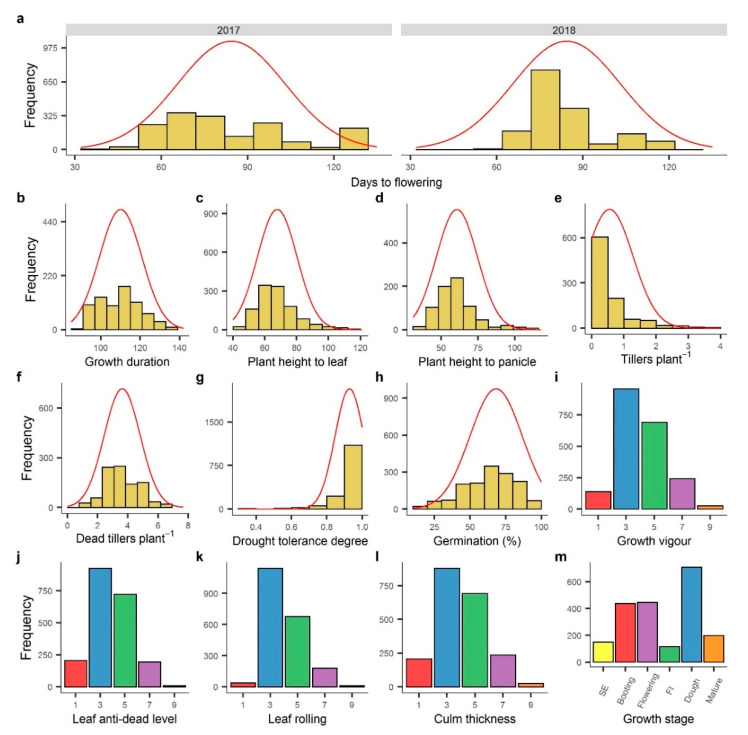
Frequency distribution of seven quantitatively measured and six visually scored traits: (**a**) days to flowering for 2017 and 2018; (**b**) growth duration (days); (**c**) plant height to leaf (cm); (**d**) plant height to panicle (cm); (**e**) tillers∙plant^−1^ (g); (**f**) dead tillers∙plant^−1^ (g); (**g**) drought tolerance degree; (**h**) germination (%); (**i**) growth vigor; (**j**) leaf anti-dead level; (**k**) leaf rolling; (**l**) culm thickness; (**m**) growth stage.

Not only were controls exhibiting significant differences, but so were populations, as shown in Figure 2. There was a wide range of phenotypic variation among the germplasm, for all the measured traits. Differences among conditions are obvious in the comparative population histograms together with the density curve. Among skewness and kurtosis, the majority of the traits were within the value of a normal distribution, while the distribution of accessions in days to flowering was somewhat flat in 2017 in comparison with that in 2018 when the majority of the germplasm showed 70 to 90 days to flowering. The growth duration trend was similar to that of days to flowering in 2017. Plant height to leaf and panicle was distributed among genotypes slightly similar to most genotypes, which expressed 50 cm to 80 cm of the mentioned trait. Less variation was observed for drought tolerance degree (DTD), yet most of the visually scored traits displayed a normal distribution of differences. Most accessions fell on scale 3 followed by scale 5 for growth duration, leaf anti-dead level, leaf rolling, and culm thickness, showing that there was genetic potential among genotypes for the water-deprived condition. Concerning the growth stage at harvest time, most of the genotypes were at the dough stage followed by the flowering and booting stage. The population of booting and stem elongation stage showed that the noteworthy germplasm never flowered, even at the end of the growing season.

A wide range of variation among the 2030 rice genotypes was observed for all the traits (Table 3). The highest coefficient of variation was depicted by tillers∙plant^−1^ (46.92%), followed by culm thickness (43.52%), leaf anti-dead level (41.64%), and growth vigor (40.83%). Drought tolerance degree was attributed the least *CV* of 8.8%. The variation range regarding the standard deviation was 0.08 (drought tolerance degree) to 22.88 (days to flowering). After days to flowering, germination percentage expressed the highest standard deviation of 18.39, followed by plant height to panicle (13.93), plant height to leaf (12.43), and growth duration (10.75).

### 2.4. Correlational Studies and Chemometric Analysis of the Germplasm

**Figure 3:**
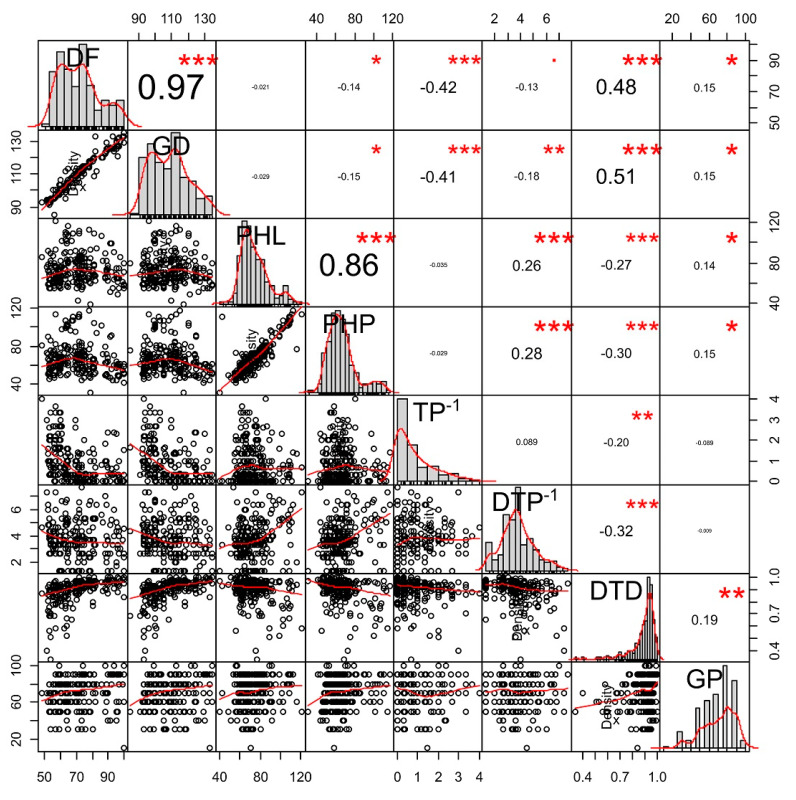
Relationship among days to flowering (DF), growth duration (GD; days), plant height to leaf (PHL; cm), plant height to panicle (PHP; cm), tillers∙plant^−1^ (TP^−1^), Dead tillers∙plant^−1^ (DTP^−1^), drought tolerance degree (DTD), and germination percentage (GP) under continuous drought stress. * *p* < 0.05, ** *p* < 0.01, *** *p* < 0.001.

A correlation matrix was used for the determination of the inter-relationship of phenology, plant height, tillers∙plant^−1^, dead tillers∙plant^−1^, drought tolerance degree, and germination, along with significance based on *p*-value (Figure 3). Most of the traits depicted highly significant relationships among themselves. Higher tillers among genotypes were due to fewer days to flowering, while, in contrast, there was higher drought tolerance degree with a higher number of days to flowering. The genotype which had longer growth duration also had more time to flowering. As the value of drought tolerance degree increased, so did growth duration and germination, while plant height, tillers∙plant^−1^, and dead tillers∙plant^−1^ decreased. Plant height to leaf depicted a significant relationship with plant height to panicle and dead tillers∙plant^−1^. Plants which had a taller height to panicle showed a higher number of dead tillers and germination percentage.

Principal component analysis (PCA) for rice accession was performed under severe conditions of drought to establish any clustering based on eight morphological traits. PCA generated a new set of eight orthogonal variables (principal components (PCs)). PCs with more than one eigenvalue were considered for interpretation [17]. For cleaner interpretation, data were subjected to Varimax orthogonal rotation. The first rotated factor (PC1) explained about 35.49% of the total variability. The second and third rotated factors (PC2 and PC3) explained about 24.77% and 12.29% of the total variability, respectively (Table 4). The variables and attributes in each principal component (PC) were skipped based on significant factor loading values less than or equal to 0.1. PC1 was positively contributed to by days to flowering, growth duration, and drought tolerance degree, while PC2 was positively contributed to by days to flowering, growth duration, plant height to leaf, plant height to panicle, and drought tolerance degree.

### 2.5. Agglomerative Hierarchical Clustering

**Figure 4:**
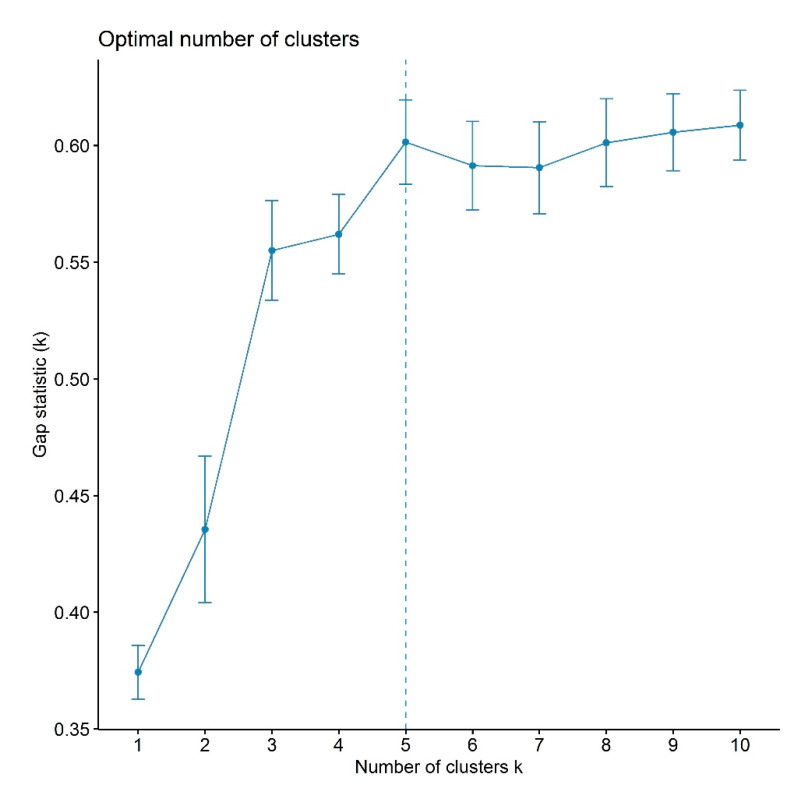
The optimal number of clusters for agglomerative hierarchical cluster (AHC) analysis.

Employing days to flowering for 2017 and 2018, a distance matrix was computed with the “Euclidean” method. Cluster analysis was performed using the “complete” method that brought about 10 clusters (Figure 4 and Figure S1, Supplementary Materials). Cluster VI was the largest cluster, comprising 311 genotypes in total, while, contrary to this, cluster X had only one accession. Clusters were sorted according to their performance in phenology against severe drought stress. The summary of cluster analysis is given in Table 5, and geographic distribution is given in Table 6. Mean longest duration from sowing to flowering was observed in cluster IV, followed by cluster V. Furthermore, those clusters expressed 0% maturity for the germplasm under study. On the other hand, although cluster X had shorter days to flowering, it resulted in the longest duration from flowering to maturity (even not matured at harvesting). Therefore, clusters IV, V, and X were categorized as worse in performance against stress and marked as highly drought-susceptible clusters. Clusters II and III were attributed with bad performance due to longer duration for days to heading and medium plant height and indicated as drought-susceptible clusters. Almost half of the population in cluster I was matured at harvesting time with plant height to leaf greater than seen for clusters II, III, IV, V, and X. It was classified as a middle group with general performance, identified as a moderately drought-resistant cluster. Mean time to flowering for clusters VI and VII was around 76 days with evident plant height. Additionally, more than 50% of accessions were matured at harvesting time; thus, those clusters could be a better choice for selection with passably drought-resistant performance. However, for the selection of genotypes with best performance in the water-deficit environment, cluster VIII should be considered. That cluster was accredited with shorter time to flowering in mean performance (almost 67 days) with obvious mean plant height to leaf. Furthermore, the matured germplasm of the cluster was around 75%; thus, that cluster was tagged as drought-resistant. Finally, we considered cluster IX as excellent among all clusters due to outstanding performance in the measured traits. It was also top-ranked regarding maturity percentage (78.72%) and average plant height to leaf and panicle (74.94 cm and 67.35 cm, respectively). That cluster revealed the shortest mean days to flowering (62.09) and growth duration (<100 days), and it was labeled as a highly drought-resistant cluster.

### 2.6. Eco-Geographic Distribution of Clusters and Final Selection

To optimize the results, ecological distribution is discussed below. Regarding geography (Table 6), there were 31 and 44 genotypes from Japan and South Korea correspondingly subjected to cluster analysis. Most of the variants of the germplasm from these countries fell in clusters VI and VII, while only one accession from South Korea was distributed in cluster VIII. Cluster X had only one genotype from South Korea, while, being the largest group, cluster VI had 97, 70, 61, 19, and 64 accessions from Jilin, Liaoning, Ningxia, Heilongjiang, and other provinces in that order. Despite being the biggest group, there were no genotypes from Anhui, Guizhou, and Henan. There was only one genotype from each of Anhui and Guizhou, and both were classified in cluster IV, led by Tianjin containing two genotypes (distributed in cluster III and V). Henan and Jiangsu province expressed the worst performance against drought with 89% and 88% of their germplasm falling in highly drought-susceptible clusters (IV and V). Shandong followed Jiangsu province in performance with 64% of germplasm distributed in highly susceptible clusters. China Agricultural University (CAU), Beijing was identified as a good performer against drought with 60% of germplasm in clusters VI and VII, and 25% in cluster I. The order of best-performing regions against severe drought stress with their collective population (%) in clusters VIII and IX was as follows: Heilongjiang (73%), Jilin (27%), Hebei (21%), and Xinjiang (17%).

There were 235 genotypes in total in the drought-resistant cluster (VIII) and highly drought-resistant cluster (IX). That number is still quite big to be considered as the final selection. Although there were vibrant differences regarding days to flowering among clusters, there was some variation among genotypes of the same cluster. Therefore, we further selected seven genotypes (five from cluster VIII and two from cluster IX) which were also coherent with another study (unpublished work). The performance of those final selected accessions was also compared to the three controls (Table 7). The selected germplasm was distinctive from controls with shorter time to flowering. The genotypes labeled as Longjing 12, Longdun 102, Yanjing 22, Liaojing 27, Xiaohongbandao, Songjing 17, and Zaoshuqingsen could be a better resource for continuous and terminal drought stress.

## 3. Discussion

The evolutionary success of annual plants depends largely on their successful tolerance to environmental stresses. At the reproductive stage, terminal drought stress alters the physiological mechanism which ultimately affects rice crop yield. Drought stress at the post-anthesis stage probably decreases the yield of grain in all genotypes due to reduced growth vigor. Previous studies showed that plants use different strategies to respond to drought stress, including drought escape (DE) [18]. DE helps plants to accelerate growth and transition quickly to the reproductive stage before the damage is irreversible to complete their life cycle. While, in rice, several plant breeders screened rice genotypes for drought tolerance based on indices of yield susceptibility to stress [19,20], we found very little information on screening drought-tolerant rice genotypes for multiple agronomic traits under continuous drought stress environments. In the present study, days to flowering were primarily used to screen the rice genotypes under drought stress conditions with other supporting agronomic traits. The agronomic traits like phenology, plant height, number of tillers∙plant^−1^, drought tolerance degree, leaf anti-dead level, germination percentage, and leaf rolling were successfully utilized under breeding programs to screen rice genotypes for their performance under drought stress conditions [14,16,21,22,23,24]. Additionally, dead tillers∙plant^−1^, culm thickness, growth vigor and stage, and maturity (%) were utilized as orientation for screening. Therefore, field measurements of the above-mentioned traits could be employed to screen a large number of genotypes in the field for rice breeding programs.

Our correlation analysis showed a positive association of days to flowering with growth duration (0.97) and drought tolerance degree (0.48), and a negative relationship with tillers∙plant^−1^ (−0.42) which were significant at the *p* < 0.01 level (Figure 3). However, days to flowering did not show a statistically significant correlation with plant height to leaf and dead tillers∙plant^−1^ under drought stress conditions. This relationship analysis was useful when screening on the basis of secondary traits. However, the association of traits revealed the level of the relationship between only two agronomic traits at a time. To deduce information from more than two physiological traits, multivariate analysis methods such as PCA and cluster analysis were performed [25]. The cluster analysis based on agronomic traits split up genotypes into 10 clusters. Genotypes in cluster VIII and IX had a shorter time to flowering and taller plant height which could be utilized as predictors of higher biomass performance under drought stress, such as better above-ground plant biomass, plant grain yield, and harvest index. Thus, those clusters were designated as drought-resistant and highly drought-resistant, respectively (Table 5). It is widely reported that there are increased days to maturity [26], as well as a reduction in germination (%) [22] and tillers∙plant^−1^, due to the scarcity of water, and this can be measured in term of maturity (%). Maturity (%), as an indicator of drought tolerance ability, was also higher in genotypes of cluster VIII and cluster IX under drought stress conditions.

An effect of an increase in days to flowering and maturity under continuous drought was seen on plant height to leaf and plant height to panicle. Lower plant height in sensitive genotypes may be due to a longer exposure to drought based on longer phenology. On the contrary, drought severity increased with an increase in the number of SMW (Figure 1), resulting in lower volumetric soil moisture content; thus, genotypes with a shorter time to flowering (cluster VIII and IX) expressed higher plant height. Although this is not always the case, genotypes susceptible to drought exhibit decreased plant height under water-deficit conditions, as supported by many previous experiments [21,24]. Ecotypes of plants play a pivotal role in the variation of time to flowering under stress [27]. Due to exclusively retaining the lowland ecotype, there were no exotic (Japan and South Korea) genotypes in drought-resistant clusters (IX and VIII) and only one from South Korea in cluster VIII. Therefore, most variants of the germplasm from these countries were moderate to fair in performance. The case was worse for the germplasm from the south of China (Jiangsu, Anhui, Henan, Guizhou, and Yunnan) with no single genotype in the drought-resistant and highly drought-resistant clusters due to possessing no upland ecotypes. This was also accredited to the long growth duration of the germplasm from those provinces of southern China [28].

It was reported that genotypes with lowland ecotypes perform well against drought as compared to upland ecotypes [29]; however, on average, upland germplasms are better against drought. As denoted by cluster analysis, the majority of lowland genotypes from Shandong (43) were sorted in clusters IV and V (highly susceptible to drought), whereas those of Liaoning were put in susceptible (109) and passably drought-resistant clusters (64). On the other hand, most of the traditional lowland accessions from Hebei (13) were in drought-resistant clusters (VI, VII, VIII, and IX). Furthermore, in central China (Beijing, Tianjin, Hebei, Liaoning, Ningxia, and Shandong), there were only eight genotypes in the highly drought-resistant cluster (IX) comprising upland (three from Liaoning) and traditional lowland (four from Ningxia and one from Hebei) ecotypes. Overall, from that region, upland ecotypes were mostly distributed among drought-resistant clusters and lowland ecotypes were dispersed in medium to bad clusters except for Liaoning (a substantial portion with better performance) and Shandong (most of the germplasm in the worst clusters). The strange phenomenon of the ecotypic distribution of north China (Heilongjiang, Jilin, Inner Mongolia, and Xinjiang) resulted in the best performance of lowland ecotypes against continuous drought. In total, 180 genotypes (43.69%) from north China were categorized in clusters VIII and IX, which was attributed to the sensitivity of germplasm to the high temperature of Beijing at the vegetative phase. This analysis unveiled the potential of the germplasm of that region against continuous drought stress. Furthermore, Wang et al. [30] found that a rice grain yield of 8 t∙ha^−1^ and even higher could be accomplished using high-yielding upland cultivars with suitable management practices in northern China. Furthermore, no genotypes were classified in clusters with susceptible and highly susceptible performance against drought except for four lowland genotypes in cluster III (two each from Jilin and Xinjiang). Hence, while selecting against drought, breeders should also focus on environmental adaptation and the ecology of respective crop plants. 

## 4. Materials and Methods

### 4.1. Experimental Site and Plant Material

**Figure 5:**
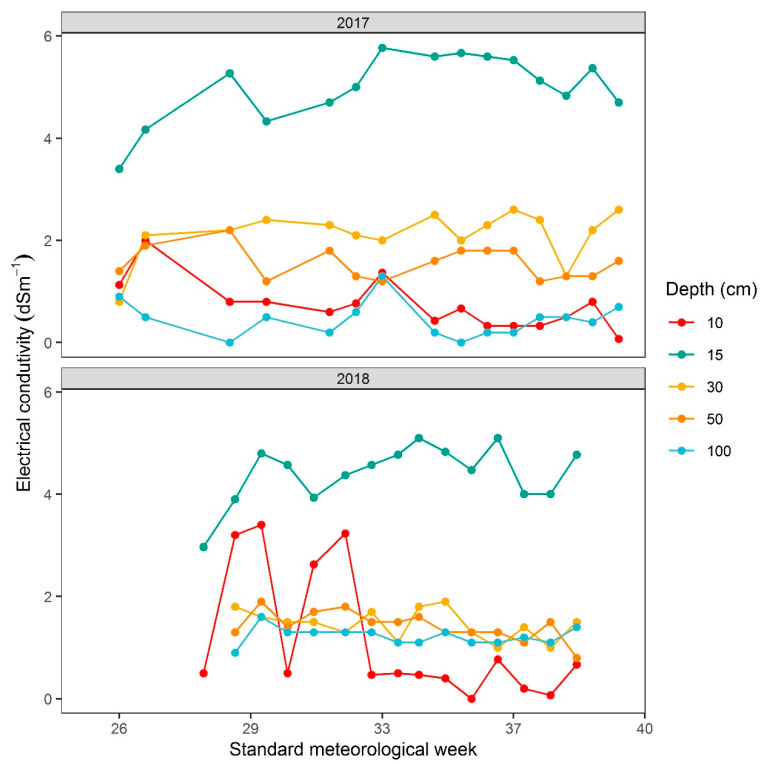
Electrical conductivity (*EC*) of soil at Shangzhuang Agricultural Research Station, Beijing, China from May to October for two consecutive trials in 2017 and 2018. Soil *EC* was recorded at 10 cm, 15 cm, 30 cm, 50 cm, and 100 cm.

**Figure 6:**
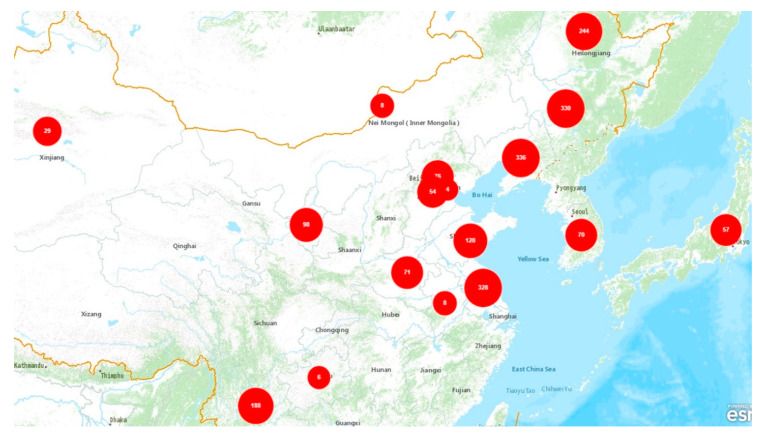
Geographic origin of accessions tested. Ivory Coast and Brazil were skipped due to thempossessing three genotypes in total.

The field experiments for the identification of drought-resistant genotypes were conducted in the summer and autumn of 2017 and 2018 at Shangzhuang Agricultural Research Station of China Agricultural University (CAU), Beijing, China (40°08’13.4” north (N), 116°11’06.6” east (E)). Meteorological observations and soil conditions during the crop period (May to October) of the two consecutive trials are presented in Figure 1 and Figure 5. The total germplasm included 2030 accessions, of which 2000 genotypes were provided by the Institute of Crop Sciences, Chinese Academy of Agricultural (CAAS), Beijing, China, and the remaining 30 cultivars were supplied by the Rice Research Center at CAU, Beijing, China. Accessions in this study originated from five countries including China (1905), South Korea (70), Japan (57), Ivory Coast (two), and Brazil (one). In China, most of the entries were distributed across Liaoning (336), Jilin (330), Jiangsu (328), and Heilongjiang (244), as evident in Figure 6. All the accessions contained most of the released varieties, as well as more traditional cultivars, some promising lines, and several exotic strains, which were derived from the *japonica* subspecies (Table S1, Supplementary Materials). These breeding lines, along with three control varieties, i.e., B_1_, Handao 277 (both tolerant controls), and 297-28 (susceptible control) were evaluated under non-stress (lowland) and drought (upland) conditions. Handao 277 is an approved upland variety cultivated by farmers in the Huai and Yellow (Huang) River basins of China. This variety was also used as a control genotype in China National Upland Rice Variety Regional Trials in the same river basin areas from 2004 to 2019. B_1_ is the most promising drought-resistant semi-dwarf mutant derived from IAPAR9 by γ-ray radiation. It shows stronger drought resistance than the wild type. While 297-28 is a drought-sensitive mutant screened from a γ-ray-mutagenized Handao 297 population, it shows thinner stems, leaves, and roots, as well as a higher number of tillers, than Handao 297 under non-stressed conditions. Furthermore, under drought stress, the leaves wilt easily, leading to sharp decreases in yield.

### 4.2. Field Management

The experiments were conducted in an ARCBD, widely used to phenotype large populations [31,32,33,34] in the field environment under a movable rainout shelter. In both years of study, experimental methods were the same unless mentioned otherwise. Before conducting the trials, the experimental field was leveled, and the seeds of all variants of the germplasm were treated with the recommended dose of fungicide. The trials were set up manually by dry direct seeding into the soil at a 2–3-cm depth in shallow furrows spaced 18 cm apart with estimated 30–35 seeds per accession per furrow. The row length for each furrow was 0.6 m with no specific plant-to-plant distance. In 2017, there was a control group in every 15 m^2^ of the rice crop area. A protection border was established by sowing Handao 297 around the test area, covering a total area of about 0.8 acres. In 2018, the sowing plan was modified based on the maturity period observed in 2017 and accomplished in two steps. Firstly, 215 late-maturing genotypes with two lines per accession were sown with a line spacing of 20 cm. On average, a control group was set up once in every 17 m^2^ of area, and a total of six control groups were set up. A protection border of Handao 297 was planted around the test area, occupying about 156.6 m^2^ of area. In the second step, after 21 days of sowing of the late-maturing group, the remaining genotypes were seeded with 19 control groups. Standard agronomic practices were adopted in both seasons of experiments. The base fertilizers consisted of 48 kg∙ha^−1^ of nitrogen (N), 120 kg∙ha^−1^ of P_2_O_5_, 100 kg∙ha^−1^ of K_2_O, 22.5 kg∙ha^−1^ of ZnSO_4_, and 30 kg∙ha^−1^ of FeSO_4_. Later, at the seedling stage, nitrogen was again applied via the placement method at the rate of 45 kg∙ha^−1^. Additionally, organic manure was applied at the rate of 3.75 t∙ha^−^**^1^**. Weeds were controlled by manual hoeing (twice each season) and uprooting, as well as by spraying herbicide along borders with the recommended dose.

### 4.3. Drought Experiments and Field Observations

Drought trials were conducted in field conditions under an automatic electrical movable rainout shelter to avoid rainfall-associated water accumulation during the stress period. The rainout shelter could be completely removed during sunny days and reset to cover the plants when it rained. Only one irrigation was applied immediately after sowing so that the seeds could properly germinate. No supplemental irrigation after stress initiation was provided even if the stress was very severe.

Trait evaluation was conducted at maturity before harvesting except for days to flowering and growth duration (Table 8). Observed traits were classified into two groups, i.e., quantitatively measured and visually scored. In total, 13 traits were observed of which six characteristics were scored visually, comprising germination percentage (GP), growth vigor (GV), leaf anti-dead level (LADL), leaf rolling (LR), culm thickness (CT), and growth stage (GS). The general criteria for visual scoring are mentioned in Table 9. Volumetric soil water content, soil temperature, and electrical conductivity (*EC*) were recorded through a precise moisture measurement (PMM) with a TRIME-PICO64 TDR Technology instrument (IMKO Micromodultechnik GmbH Company, Ettlingen, Germany) inserted at 10 cm, 15 cm, 30 cm, 50 cm, and 100 cm of soil depths with two fixed locations for the 100-cm depth containing pre-buried tubes for inserting measurement probes and three random locations for other depths of the stressed field.

### 4.4. Modifications for the Screening of a Large Germplasm

As the germplasm was quite big, the study was modified for the management of resources and land. For ARCBD, the control groups were planted with the same planting specifications as mentioned above for drought situations. All controls in each group were sown adjacently, and there were 24 and 28 control groups in 2017 and 2018, respectively. Each control group contained three control genotypes. In ARCBD, the number of control groups directly corresponds to the number of blocks of that experiment. To make this single replicated experiment more precise, traits were measured from three random samples within accessions to get means, except for phenology, which was measured one time per genotype. Out of the measured traits, only days to flowering were recorded in the second year of study. During these two years, the screening of 2030 rice genotypes was performed based on agronomic traits only. To avoid data imputation, correlational studies were carried out involving those 212 genotypes whose values for subjected traits were not missing. We manually adjusted the number of clusters to 10 although R software suggested it as five, as shown in Figure 4. This was deduced according to the highest value of the gap statistic. Furthermore, our germplasm was quite big, and it was rather difficult to divide into five groups only.

### 4.5. Statistical Analysis

#### 4.5.1. Phenotypic Data Analysis

Following a previously described procedure to minimize the effects of environmental variation [31,32,33,34], the two years of phenotypic data for days to flowering were fitted with a linear mixed model that included the effects of treatments, genotypes, controls, blocks, and genotypes × controls. All data were analyzed statistically and graphically using R version 3.6.2 [36]. The standard errors of the mean (± *SE*) and treatment means were compared with Tukey’s honestly significant difference (HSD) by using the agricolae [37] and augmentedRCBD [38] libraries at 5% probability. For ARCBD, the various standard errors of mean difference were computed as shown in Equations (1)–(4).

(1) $$Sc=\sqrt{\frac{2MSe}{r}},$$

(2) $$Sb=\sqrt{2MSe},$$

(3) $$Sv=\sqrt{2MSe\left(1+\frac{1}{c}\right)},$$

(4) $$Svc=\sqrt{MSe\;\left(1+\frac{1}{r}+\frac{1}{c}+\frac{1}{rc}\right)},$$

where *Sc*, *Sb*, *Sv*, and *Svc* are the difference between two controls, the difference between two test genotypes in the same block, the difference between two controls in different blocks, and the difference between control and test genotypes, respectively; *r* is the number of blocks, while c is the number of controls in the experiment. For Tukey’s HSD test, the first three standard errors (*SE*) above were multiplied by *q*(*t*, *v*; *α*), where *q* is the upper point of the studentized range at the specified level of significance (*α*) for the total number of treatment means (*t*) and the error degrees of freedom (*v*). To get an HSD value for *Svc*, q(t, v; α) was multiplied by $\sqrt{\frac{\mathrm{MSe}}{H^{\prime }}}$, where *H*′ is the harmonic mean of the coefficients for standard errors of a difference.

#### 4.5.2. Descriptive Statistics and Data Visualization

The descriptive statistics for days to flowering such as mean, standard error, minimum, maximum, skewness, and kurtosis with *p*-values from for the adjusted means from the results were computed by loading the augmentedRCBD library [38]. Using the same package, genetic variability statistics such as mean, phenotypic, genotypic and environmental variation, phenotypic, genotypic, and environmental coefficient of variation [39,40], broad-sense heritability (*h*^2^) [41], *h*^2^ category according to Robinson [42], and genetic advance (*GA*) were estimated using the gva.augmentedRCBD() function.

For all the graphs except for the agglomerative hierarchical cluster (AHC), package ggplot2 [43] was used with other packages for data visualization. R function chart.Correlation from PerformanceAnalytics [44] with the default Pearson method was used to visualize trait correlations. To draw the AHC, we loaded the cluster [45] and factoextra [46] packages. Maps in this article were created using ArcGIS^®^ software by Esri (ArcGIS^®^ and ArcMap™ are the intellectual property of Esri and are used herein under license. Copyright^©^ Esri. All rights reserved. For more information about Esri^®^ software, please visit www.esri.com).

## 5. Conclusions

Overall, it can be concluded that this study found substantial variation in the terminal drought stress tolerance among rice genotypes, and several relative drought stress-resistant and -sensitive rice genotypes were identified based on agronomic traits. We showed that certain morphological traits using statistical methods such as multivariate analysis can be used to identify a large number of genotypes for the ability to tolerate drought stress. It would be best to check the genotypes for large germplasms with agronomic, morphological, and visually graded traits. This type of screening process identifies and screens genotypes with higher overall stress tolerance, and such genotypes can also be used as parental genotypes in breeding programs to grow terminal drought-tolerant rice genotypes. The top seven genotypes labeled as Longjing 12, Longdun 102, Yanjing 22, Liaojing 27, Xiaohongbandao, Songjing 17, and Zaoshuqingsen can be better resources for continuous and terminal drought stress. However, the evaluation of time to flowering concerning physiological and molecular parameters was limited by time and resources, as it was practically difficult to evaluate such a big germplasm in laboratory experiments. Furthermore, evaluations for drought avoidance with a focus on below-ground traits are required. Lastly, there is a need to study the interaction of continuous drought stress with the specific air temperature of the greenhouse, which rises when the movable rainout shelter is above the crop as compared to the rest of the field.

## Figures and Tables

**Table 1:** Summary of the statistical analysis by augmented randomized complete block design (ARCBD) of the effects of treatments, genotypes, and controls on days to flowering in 2017 and 2018, as well as the pooled data.

Source	2017			2018			Pooled		
	*df*	*F*	*p*-Value	*df*	*F*	*p*-Value	*df*	*F*	*p*-Value
Block (ignoring treatments)	19	392.45	0.000	24	415.92	0.000	44	103.5	0.000
Treatment (eliminating blocks)	1548	13.79	0.000	1323	2.96	0.000	1726	4.1	0.000
Control	2	42.44	0.000	2	7.54	0.001	2	11.28	0.000
Block (eliminating treatments)	19	1.77	0.070	24	1.78	0.030	44	9.66	0.000
Treatment (ignoring Blocks)	1548	18.59	0.000	1323	10.47	0.000	1726	6.5	0.000
Genotype	1545	18.57	0.000	1320	10.43	0.000	1723	6.48	0.000
Treatment: test vs. control	1	2.02	0.160	1	68.6	0.000	1	28.72	0.000
Residuals	38			72			1255		

*df* = degrees of freedom.

**Table 2:** Descriptive statistics for days to flowering in 2017 and 2018, as well as pooled conditions, for treatments, genotypes, and controls (B₁, Handao 277, and 297-28).

Factor	*M*	*SE_M_*	*SD*	Min	Max	Kurtosis	Skewness	*CV* (%)	*H*^2^ (%)	*GA* (%)	*SE* cate.	HSD_0.05_
Pooled												
Treatment	84.34	0.45	18.80	50	133	−0.301	0.768	21.83				
Genotype	84.46	0.36	19.09	50	133	0.096	0.762	22.60	84.56	40.21	*Sv*	53.544
Control	81.76	0.60	6.98	56	105	1.869	0.538	8.54	39.47	5.78	*Sc*	10.393
B₁	82.84	0.88	5.90	71	105	6.331	2.070				*Sb*	46.372
Handao 277	76.78	0.65	4.36	56	82	11.548	−2.794				*Svc*	27.363
297-28	85.67	1.08	7.23	72	100	−1.284	−0.032					
2017												
Treatment	84.47	0.57	22.88	50	133	−0.568	0.638	27.09				
Genotype	84.51	0.59	23.25	50	133	−0.640	0.627	27.52	94.61	45.33	*Sv*	36.976
Control	83.50	1.14	8.788	56	105	0.893	−0.001	10.53	67.45	13.14	*Sc*	6.391
B₁	84	1.79	8.01	75	105	2.376	1.807				*Sb*	32.068
Handao 277	75.40	1.27	5.68	56	80	6.996	−2.445				*Svc*	18.806
297-28	91.10	0.82	3.66	87	100	0.499	1.032					
2018												
Treatment	84.2	0.33	12.46	53	116	0.598	1.162	14.80				
Genotype	84.41	0.35	12.72	53	116	0.414	1.108	15.07	90.42	23.89	*Sv*	80.668
Control	80.37	0.54	4.71	71	94	1.346	0.954	5.86	21.35	1.94	*Sc*	10.404
B₁	81.92	0.67	3.31	71	87	4.337	−1.818				*Sb*	69.883
Handao 277	77.9	0.511	2.56	71	82	1.128	−0.181				*Svc*	40.663
297-28	81.32	1.28	6.40	72	94	−0.284	1.064					

*M* = mean, *SE_M_* = standard error of the mean, *SD* = standard deviation, *CV* = coefficient of variation, *H*² = heritability in broad sense, *GA* = genetic advance, *SE* cate. = standard error category, HSD_0.05_ = Tukey’s honestly significant difference at 5% level of significance, *Sv* = difference between two test genotypes in different blocks, *Sc* = difference between two controls, *Sb* = difference between two test genotypes in the same block, *Svc* = difference between control and test entry.

**Table 3:** Descriptive statistics for traits measured and visually scored in the study.

Trait	*M*	*SE_M_*	*SD*	Min	Max	Range	Median	Mode	*CV* (%)	*s* ^2^	Kurtosis	Skewness
DF	84.47	0.571	22.88	50	133	83	78	71	27.09	523.54	−0.57	0.64
GD (days)	110.08	0.403	10.75	85	142	57	110	100	9.77	115.56	−0.63	0.23
PHL (cm)	67.77	0.347	12.43	39.67	120.67	81	65.67	62.67	18.35	154.61	1.62	1.08
PHP (cm)	60.69	0.510	13.94	31.33	117.6	86.27	58.67	64	22.96	194.19	2.37	1.28
TP^−1^	1.55	0.020	0.73	1	6	5	1.33	1	46.92	0.53	4.18	1.88
DTP^−1^	3.62	0.033	1.19	0.33	7.67	7.34	3.67	3.33	32.92	1.42	0.18	0.46
DTD	0.93	0.002	0.08	0.28	1	0.72	0.95	0.97	8.80	0.01	12.52	-2.96
GP	68.23	0.406	18.39	10	100	90	70	70	26.95	338.22	−0.10	-0.51
GV	4.08	0.037	1.67	1	9	8	3	3	40.83	2.78	−0.03	0.46
LADL	3.90	0.036	1.63	1	9	8	3	3	41.64	2.64	−0.24	0.23
CT	4.01	0.039	1.75	1	9	8	3	3	43.52	3.05	−0.17	0.33
LR	4.01	0.031	1.41	1	9	8	3	3	35.21	2.00	0.29	0.82

*M* = mean, *SE_M_* = standard error of the mean, *CV* = coefficient of variation, *s*^2^ = variance, DF = days to flowering, GD = growth duration, PHL = plant height to leaf, PHP = plant height to panicle, TP^−1^ = tillers plant^−1^, DTP^−1^ = dead tillers plant^−1^, DTD = drought tolerance degree, GP = germination (%), GV = growth vigor, LADL = leaf anti-dead level, CT = culm thickness, LR = leaf rolling, GS = growth stage.

**Table 4:** Varimax-rotated factor loadings of the significant principal components (PCs).

Variables	PC1	PC2	PC3	PC4	PC5	PC6	PC7	PC8
Days to flowering	0.499	0.278	0.201	0.144	0.218	0.262	0.101	0.697
Growth duration (days)	0.510	0.263	0.172	0.105	0.240	0.246		−0.714
Plant height to leaf (cm)	−0.238	0.592		−0.228	0.229		−0.696	
Plant height to panicle (cm)	−0.291	0.560		−0.237	0.125	−0.160	0.707	
Tillers∙plant^−1^	−0.283	−0.264	−0.286	0.261	0.817	0.175		
Dead tillers∙plant^−1^	−0.256	0.218	0.338	0.825	−0.101	−0.283		
Drought tolerance degree	0.440		−0.283		0.219	−0.822		
Germination (%)		0.266	−0.806	0.333	−0.318	0.239		
Eigen value	2.840	1.981	0.983	0.823	0.724	0.494	0.129	0.026
Variability (%)	35.49	24.77	12.29	10.29	9.045	6.177	1.614	0.327
Cumulative (%)	35.49	60.26	72.55	82.84	91.882	98.059	99.673	100

Note. Principal component values less than 0.1 were skipped.

**Table 5:** Clusters accompanied by their size, mean, minimum, maximum, and performance against drought stress of different characteristics with general performance against severe drought.

Cluster	Count	Statistic	DF (2017)	DF (2018)	DF (pool.)	GD	Mat (%)	PHL	PHP	Performance
**I**	127	*M*	81.94	85.72	84.08	119.07	46.46	67.25	57.01	Moderately drought-resistant
		Min	68	81	79	108		46	42.33	
		Max	93	99	92	132		99.2	94.67	
**II**	36	*M*	100.25	96.17	98.44	129.25	11.11	61.41	49.58	Drought-susceptible
		Min	90	91	96	127		50.67	37	
		Max	106	106	104	132		77	66.67	
**III**	100	*M*	99.62	83.83	92.00	130.53	15	63.59	51.70	
		Min	93	76	86	121		41	39.33	
		Max	110	92	99	135		92	79.67	
**IV**	104	*M*	127.98	110.84	119.64		0	61.77	58.43	Highly drought-susceptible
		Min	114	104	111	N		47	31.67	
		Max	133	116	124			113.67	110.33	
**V**	27	*M*	102.56	109.81	106.44	N	0	60.58	49.48	
		Min	100	107	104			50.67	43	
		Max	110	116	112			73.67	58.33	
**VI**	311	*M*	73.71	77.60	75.92	112.96	59.16	71.60	65.00	Passably drought-resistant
		Min	64	68	68	95		39.67	31.33	
		Max	93	82	86	137		111.33	113	
**VII**	131	*M*	68.06	83.73	76.15	107.35	52.67	69.30	61.46	
		Min	57	80	70	95		47.33	42.33	
		Max	80	89	84	118		99	100	
**VIII**	188	*M*	59.14	74.52	67.10	99.01	75.53	72.01	65.30	Drought-resistant
		Min	51	69	62	85		48	38.67	
		Max	66	81	71	113		120.67	114	
**IX**	47	*M*	57.62	66.06	62.09	99.30	78.72	74.94	67.35	Highly drought-resistant
		Min	50	60	58	87		57.67	49.82	
		Max	69	69	67	110		102.6	98.6	
**X**	1		75	53	64		0	65.67	53	Highly drought-susceptible
***SD***			21.31	17.18	18.27	12.06	30.36	4.77	6.41	
***SE_M_***			6.74	5.43	5.78	4.56	9.60	1.51	2.03	

DF = days to flowering, pool. = pooled, GD = growth duration, Mat = maturity, PHL = plant height to leaf, PHP = plant height to panicle, *M* = mean, N = not matured, *SD* = standard deviation, *SE_M_* = standard error of the mean.

**Table 6:** Geographic distribution of clusters among Japan, South Korea, and various regions of China.

	Cluster	I	II	III	IV	V	VI	VII	VIII	IX	X	Total	*SD*	*SE_M_*
Origin														
Anhui					1							1	0.00	0.00
Beijing		5	3	6			4		2			20	1.41	0.63
CAU, Beijing		4		3			8	2				17	2.28	1.14
Guizhou					1							1	0.00	0.00
Hebei		3	5	8	1		7	2	6	1		33	2.57	0.91
Heilongjiang		2					19	23	88	31		163	29.28	13.09
Henan		1	1	1	16	10						29	6.18	2.76
Inner Mongolia		2						2	4			8	0.94	0.54
Japan		10	1	8			7	5				31	3.06	1.37
Jiangsu		1	1	3	51	5	3					64	18.09	7.38
Jilin		16		2			97	64	62	5		246	35.47	14.48
Liaoning		54	13	46	1		70	20	12	3		219	24.13	8.53
Ningxia		9					61	3	9	4		86	22.04	9.86
Shandong		3	5	4	33	11	12	1				69	10.18	3.85
South Korea		7	5	8		1	15	6	1		1	44	4.47	1.58
Tianjin				1			1					2	0.00	0.00
Xinjiang		2		2			6	3	4	3		20	1.37	0.56
Yunnan		8	2	8			1					19	3.27	1.63
Total		127	36	100	104	27	311	131	188	47	1		86.88	27.48

*SD* = standard deviation, *SE_M_* = standard error of means, CAU = China Agricultural University, Beijing.

**Table 7:** Top seven accessions along with the control group for a primary trait of selection (days to flowering) and supporting characters (TP**^−1^**, DTP**^−1^**, LADL, LR, GS).

Cluster	Accession Code	Name	DF17	DF18	DF	TP^−1^	DTP^−1^	LADL	LR	GS
VIII	2017-G0100	Longjing 12	56	76	66	-	-	3	5	M
VIII	2017-G0319	Longdun 102	60	70	65	3	3	5	3	D
VIII	2017-G0376	Yanjing 22	58	76	67	2	2	5	5	D
VIII	2017-G0607	Liaojing 27	65	76	71	1.33	6	5	5	D
VIII	2017-G0710	Xiaohongbandao	52	73	63	4	5.33	3	3	D
IX	2017-G0027	Songjing 17	52	67	60	-	-	5	3	M
IX	2017-G0178	Zaoshuqingsen	62	68	65	-	-	5	5	M
VI		Handao 277	75	77.88	77	1	1	3	4	FI
I		B₁	84	81.92	83	1	4	2	3	D
I		297-28	91	82.31	87	1	3	3	3	F

Note. Accession identification was converted to an accession code for the sake of secrecy. The name represents the exact genotype. DF17 = days to flowering in 2017, DF18 = days to flowering in 2018, DF = days to flowering, TP**^−1^** = tillers∙plant**^−1^**, DTP**^−1^** = dead tillers∙plant**^−1^**, LADL = leaf anti-dead level, LR = leaf rolling, GS = growth stage, M = mature, D = dough, FI = filling initiate, F= flowering.

**Table 8:** Traits measured in this study. The first seven traits were quantitatively measured parameters, while the rest were scored visually.

Trait	Description
DF	Days to flowering was recorded as the number of days from sowing to the time when inflorescences emerged above the flag leaf sheath for more than half of the individuals of a landrace.
GD (days)	Days to maturity was recorded as the number of days from sowing to the time when inflorescences ripened for more than half of the individuals of a landrace.
PHL (cm)	Plant height to leaf was measured as the height from ground to the highest leaf tip with a meter rod.
PHP (cm)	Plant height to panicle was measured as the height from ground to panicle tip with a meter rod.
TP^−1^	Tiller number was evaluated by manual counting when grains fully ripened.
DTP^−1^	Dead tiller number was evaluated when grains fully ripened.
DTD	Drought tolerance degree was measured as the mean of the ratios of green leaf length to total leaf length of the top three leaves in every plant after severe drought treatment.
GP	Germination was measured as the percentage ratio of germinated plants to total number of grains sown.
GV	Overall growth vigor of the accession was visually measured.
LADL	Leaf anti-dead level represents an estimate of the ratio of green leaf to dry leaf, which was visually measured unlike DTD.
CT	Culm thickness indicates the rice plant stem width from best (1) to worst (9).
LR	Leaf rolling was recorded to represent the leaf folding level in genotypes.
GS	Growth stage at harvesting was categorized into six stages as shown in Figure 4.

DF = days to flowering, GD = growth duration, PHL = plant height to leaf, PHP = plant height to panicle, TP^−^^^−1^^ = tillers∙plant^−^^^−1^^, DTP^−^^^−1^^ = dead tillers∙plant^−^^^−1^^, DTD = drought tolerance degree, GP = germination (%), GV = growth vigor, LADL = leaf anti-dead level, CT = culm thickness, LR = leaf rolling, GS = growth stage.

**Table 9:** General scale used in standard evaluation system (SES) for rice [35].

Index Value	General Description and Desirability		For Stress	
		Judgment	Severity of Incidence(Factual) ^a^	Code Symbol
**Blank**	No data or missing point			Blank
0	Absence of trait, no visible symptom or injury	Similar to the best resistant control, good	0%	HR
1				HR
2	Trait expression is satisfactory (useful) from the plant breeder’s point of view and the parent of variety can be used as a donor		Less than 5%	R
3				MR
4	Trait expression is not as good as it should be but may be acceptable under some circumstances (i.e., quantitative resistance under low or intermediate disease pressure)	Between resistant and susceptible control, fair	6% to 25%	MS (Intermediate)
5				
6				
7	Trait expression is unsatisfactory (not useful) in terms of commercial acceptability or genetic improvement of a crop	Similar to most susceptible control, poor	More than 25%	S
8				
9				HS

^a^ Intensity may vary depending upon the type of stress. HR = highly resistant reaction, R = resistant reaction, MR = moderately resistant reaction, MS = moderately susceptible reaction, S = susceptible reaction, HS = highly susceptible reaction.

## Supplementary Materials

The following are available online at https://www.mdpi.com/2223-7747/9/5/609/s1, Figure S1: Agglomerative hierarchical cluster analysis (AHC) of rice genotypes centered on days to flowering in 2017 and 2018. Table S1. Rice germplasm background information.
